# The mitochondrial genome of *Hilara* sp. (Diptera: Empididae)

**DOI:** 10.1080/23802359.2019.1644563

**Published:** 2019-07-23

**Authors:** Shang Gao, Hui Dong, Ding Yang

**Affiliations:** a College of Plant Protection, China Agricultural University, Beijing, China;; b Key Laboratory of Southern Subtropical Plant Diversity, Fairylake Botanical Garden, Shenzhen and Chinese Academy of Sciences, Shenzhen, China

**Keywords:** Mitochondrial genome, Empidinae, phylogenetics

## Abstract

The dance fly *Hilara* sp. belongs to the subfamily Empidinae of Empididae. The mitogenome (GenBank accession number: MN064659) of *Hilara* sp. was sequenced, the new representative of the mitogenome of the subfamily. The nearly complete mitogenome is 14,927 bp totally, consisting of 13 protein-coding genes, 2 rRNAs, and 22 transfer RNAs. All genes have the similar locations and strands with that of other published species of Empididae. The nucleotide composition biases towards A and T, which together made up 75.7％of the entirety. Bayesian inference analysis strongly supported the monophyly of Empidoidea, Empididae, and Dolichopodidae. It is clear that the phylogenetic relationship within Empidoidea: Dolichopodinae was the sister group to Neurigoninae, and Empidinae was the sister group to Trichopezinae, and the clade that contains Dolichopodinae and Neurigoninae was assigned to the sister group to the clade that contains Empidinae and Trichopezinae.

## Introduction

Empididae is one of the largest families in Diptera with over 5000 described species from the world (Yang et al. 2007). They capture aphids, psyllids, and coccids of Hemiptera, but also other true flies such as agromyzid flies, mosquitos, blackflies, and so on. They are widely used as a biological indicator of evaluating the quality of environment and biodiversity (Yang and Yang 2004).

The specimens of *Hilara* sp. used for this study were collected in Xinglong County of Hebei by Liang Wang and identified by Ding Yang. Specimens are deposited in the Entomological Museum of China Agricultural University (CAU) with the accession number CAUYD3011 (Room 2005, Plant Protection Building, West Campus, China Agricultural University). The total genomic DNA was extracted from the whole body (except the head) of the specimen using the QIAamp DNA Blood Mini Kit (Qiagen, Germany) and stored at −20 °C until needed. The mitogenome was amplified and sequenced as described in our previous study (Wang, Li, et al. 2016). The nearly complete mitogenome of *Hilara* sp. is 14,927 bp (GenBank accession number: MN064659). It encoded 13 PCGs, 22 tRNA genes and 2 rRNA genes and were similar with related reports before (Li et al. 2016; Wang, Ding, et al. 2016; Wang, Wang, et al. 2016; Li et al. 2017; Zhou et al. 2017; Qilemoge et al. 2018; Gao et al. 2018). All genes have similar locations and strands with that of other published Empididae species. The nucleotide composition of the mitogenome was biased toward A and T, with 75.7% of A + T content (A = 38.8%, T = 36.9%, C = 14.7%, G = 9.6%). The A + T content of PCGs, tRNAs, and rRNAs is 74.4, 76.7, and 80.8%, respectively. The total length of all 13 PCGs of *Hilara* sp. is 11,126 bp. Six PCGs (*NAD2*, *ATP8, NAD3, NAD5, NAD6,* and *NAD1*) initiated with ATT codons, and six PCGs (*COII*, *COIII*, *ATP6*, *NAD4*, *NAD4L,* and *CYTB*) initiated with ATG codons, and *COI* initiated with TCG as a start codon. Ten PCGs used the typical termination codons TAA except *COII* and *NAD5* used T and *NAD4* used TAG in *Hilara* sp.

**Figure 1.**
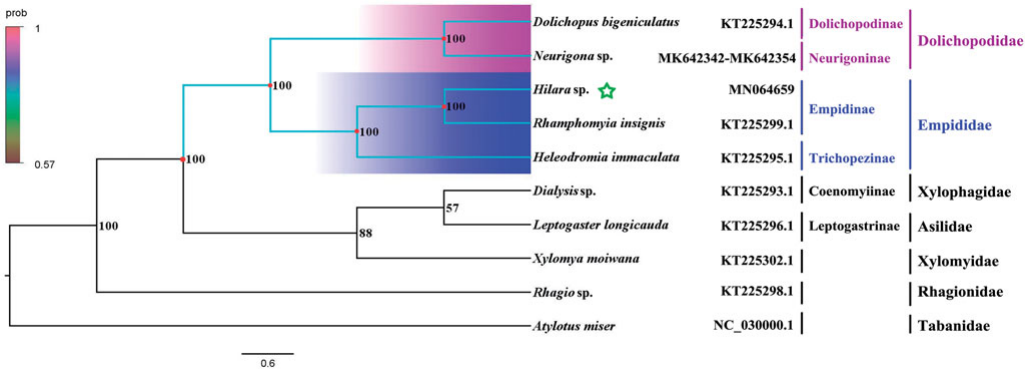
Bayesian phylogenetic tree of 10 Diptera species. The posterior probabilities are labeled at each node. Genbank accession numbers of all sequence used in the phylogenetic tree have been included in Figure 1 and corresponding to the names of all species.

Phylogenetic analysis was performed based on the nucleotide sequences of 13 PCGs from 10 Diptera species. Bayesian (BI) analysis generated the phylogenetic tree topologies based on the PCGs matrices (Figure 1). The phylogenetic result shows that the monophyly of Empidoidea, Dolichopodidae, and Empididae were strongly supported. The monophyletic Dolichopodidae that contains Dolichopodinae and Neurigoninae was assigned to the sister group to the clade of Empididae that consists of Empidinae and Trichopezinae in this study. It is clear that the phylogenetic relationship within Empidoidea: Dolichopodinae was the sister group to Neurigoninae, and Empidinae was the sister group to Trichopezinae, and the clade that contains Dolichopodinae and Neurigoninae was assigned to the sister group to the clade that contains Empidinae and Trichopezinae. This result shows that Dolichopodidae is the sister group to Empididae, which is consistent with the phylogenetic result of the previous research (Wang, Li, et al. 2016). The mitogenome of *Hilara* sp. could provide important information for further studies of Empidoidea phylogeny.
